# Temporal changes in toe-brachial index results in haemodialysis patients

**DOI:** 10.1371/journal.pone.0301376

**Published:** 2024-04-25

**Authors:** Belinda L. Baines, Timothy Pianta, Mark Tacey, Cassandra Bramston, Matthew Cotchett, Stephen Tucker, Rebecca L. Jessup

**Affiliations:** 1 Podiatry & Orthotics Department, Northern Health, Epping, Australia; 2 Nephrology Department, Northern Health, Epping, Australia; 3 Medical Education, University of Melbourne, Melbourne, Australia; 4 Northern Centre for Health and Research (NCHER), Northern Health, Epping, Australia; 5 School of Allied Health, Human Services and Sport, La Trobe University, Melbourne, Australia; 6 Staying Well and Hospital Without Walls Program, Northern Health, Epping, Australia; 7 School of Rural Health, Monash University, Warragul, Australia; National Healthcare Group, SINGAPORE

## Abstract

**Introduction:**

Toe brachial index (TBI), the ratio of toe pressure to systolic blood pressure (SBP), helps predict peripheral arterial disease. In patients with kidney failure this may be performed during haemodialysis for convenience. Until recently there has been little evaluation of the impact of haemodialysis in limb and systemic perfusion on these values. We aimed to determine if the values of TBI would change during and after dialysis compared to pre-dialysis assessments.

**Methods:**

Using a repeated measures study, TBIs and toe pressures were measured using the Hadeco Smartop Vascular Ultrasound Doppler in 31 patients undergoing haemodialysis. TBI assessments were completed pre-dialysis and compared to values obtained at 1 hour, 2 hours, 3 hours, and post-dialysis to monitor change in TBI results. Comparison of values for each patient were tested for differences using paired t-tests. Linear mixed-effects models were used to test for the effect of patient and clinical factors on change in outcome measures.

**Results:**

Mean TBI decreased from pre-dialysis at 1 hour (0.72 to 0.63, p = 0.01) and remained lower at 2 hours and 3 hours, before returning to pre-dialysis levels at post-dialysis. Mean systolic blood pressure also declined during dialysis. Mean TBI results were lower in those with a history of lower limb ulceration and in females.

Sixteen patients (51.6%) had a normal TBI at baseline, 14 (45.2%) had a mildly low TBI, and one (3.2%) had a severely low TBI. Between baseline and 1 h, five patient’s results moved from normal to mildly abnormal and one from mildly abnormal to severely abnormal. As haemodialysis concluded (post-dialysis) there were 17 (56.7%) ‘normal’ TBIs, with no severely abnormal TBIs (p = 0.73). 0.30)

**Conclusion:**

TBI and toe pressures are impacted significantly by dialysis. TBI and toe pressure assessments should be conducted before haemodialysis begins, or between dialysis sessions to avoid variability.

## Introduction

Individuals with end stage renal disease treated with haemodialysis are at risk of lower limb ulceration and amputation [1], the greatest risk factors for ulceration include a history of prior ulceration or amputation and diabetes [2], peripheral arterial disease (PAD) (affecting 18%-31% of patients treated with haemodialysis) [3]), and neuropathy [4]. For patients with diabetes and kidney failure the risk of amputation is approximately ten-fold the risk for those with just diabetes and nine times the risk for patients with kidney failure who do not have diabetes [2]. The prevalence of foot ulcers for individuals on maintenance haemodialysis increases the risk of mortality, with 7.8% of infection-related deaths relating to infection in the foot and lower limb [5].

Non-invasive investigations are utilised to complement clinical assessment [6] to provide prognostic information in patients receiving haemodialysis [7]. The assessment of systolic blood pressure at a toe–presented either as an absolute toe pressure, or divided by systemic blood pressure as a toe brachial index (TBI)—is less susceptible to artefact from medial artery calcification [6] (which can influence similar lower limb arterial tests such as ankle brachial indices (ABIs)) and is now widely advocated to detect PAD [8]. It is important that TBI results are accurate as they are used as part of the Wound/Ischemia/foot Infection (WIfI) classification system to estimate healing likelihood and amputation risk [6]. TBI values ≤ 0.3 are commonly reported to represent severe reduction in wound healing capabilities while a TBI of 0.70 or > has been reported as “normal” and is used as the threshold to make the diagnosis of PAD unlikely [8]. Similarly, an absolute toe pressure of 30 mmHg or less is reported to be indicative of severe PAD and an indication for urgent vascular imaging and revascularisation [6], while an absolute toe pressure ≥30 mmHg is reported to increase the probability of wound healing [9].

Numerous factors affect systemic pressure and limb perfusion during haemodialysis including ultrafiltration and the cooling of blood typically to 35.5 to 36.5 deg C, which results in contraction of blood vessels to maintain blood pressure, with a variable effect on cardiac output and systemic blood pressure [10, 11]. Intradialytic hypotension, a pathological fall in blood pressure experienced during haemodialysis has as estimated prevalence of 20–50% of all haemodialysis sessions—depending on definition—and occurs most commonly in the second or third hour [10, 11]. Similarly, a paradoxical increase in blood pressure during haemodialysis, intradialytic hypertension, affects approximately 18% of haemodialysis sessions [11, 12].

There is little evidence on how, or if, haemodialysis affects the measurement of toe pressures, SBP, and their ratio (TBI). A recent study by Carle et al., (2023) similarly looked to assess for any changes and investigated two time points (1 hour into dialysis and during the last 15 minutes of dialysis) and found that there was no significant change in TBI during dialysis [13].

Arterial assessments and treatment often occur opportunistically during dialysis sessions, and to date there is no evidence to suggest whether this practice is appropriate or not [14, 15]. Therefore, the aim of this study was to assess temporal changes affecting TBI results in patients with undergoing haemodialysis.

## Materials and methods

### Design

A repeated measures within-subject study design, whereby all participants received every level of treatment, was used to assess change within the same individual over time and to determine whether there was any change in TBIs in patients undergoing haemodialysis.

### Setting

The study was conducted at three dialysis sites at Northern Health (NH), a major metropolitan health service in Melbourne, Australia. Haemodialysis is provided as a day procedure.

### Recruitment and consent

Dialysis nurses identified patients meeting the inclusion criteria and who were well enough to participate on the day. Patients were further then approached by an investigator, who explained the purpose of the study, confirmed eligibility and gained informed written consent from each participant. A convenience sample was recruited from May 2021 to September 2021 and patients were eligible if they were receiving haemodialysis and admitted as same-day inpatients at the time when the principal investigator or an associate investigator were available to screen but were excluded if they had cognitive impairment or were under 18 years of age.

### Ethics

The study was approved by NH Human Research Ethics Committee (HREC/60657/NH-2020-218344). Participants were given written information and informed consent was gained prior to enrolment. Interpreters assisted with consent and support if subjects spoke a language other than English.

### Measures

The primary outcome measure was the comparison of TBI, measured at baseline (pre-dialysis) and compared at hourly intervals: during dialysis at hour one of dialysis (1 h), hour two of dialysis (2 h), and hour three of dialysis (3 h), as well as post-dialysis. All measures collected on an individual participant were collected by a single clinician. A data collection instrument was developed for clinicians to enter readings across the five time points.

### Procedure

The TBI was calculated by dividing the absolute toe pressure by the SBP on limbs located on the same side. SBP and diastolic blood pressure (DBP) were recorded at the brachial artery contralateral to any arteriovenous fistula (to avoid the risk of fistula thrombosis, and as is current practice within the satellite dialysis clinic), using a fully automated oscillometric blood pressure cuff integrated with the haemodialysis machine (Fresenius, Model 5008S). Two podiatrists collected the TBI measures at the five time points. TBI technique used by each clinician was assessed by the lead author prior to data collection to ensure method was consistent across all clinicians. Toe pressure assessments were completed by a single podiatrist during each session (two podiatrists in total) using Hadeco Smartdop Vascular Ultrasound Doppler with LCD (with Hadeco 1.9cm Toe Cuff) which automatically calculates the absolute toe pressure.

Current guidelines for assessing toe-brachial indexes recommend assessing the brachial pressure in both arms, and then using the higher brachial pressure paired with the foot pressure on the corresponding side [13]. As we were unable to compare brachial pressures in our participants (due to the presence of the AV fistula and dialysis machine tubes on one side), we determined that the best procedure would be to take the foot pressure from the side corresponding with the arm where the brachial pressure could be taken.

We used a standardised procedure to measure absolute toe pressure. The toe cuff was placed around the base of the hallux securely. The photoplethysmography (PPG) probe was placed on the distal pulp of the hallux so that the probe was flush with the skin surface, affixed with tape. A strong cyclical signal was noted from the probe to appear on the Doppler screen. The absolute toe pressure was then automatically measured by the Hadeco Smartdop Vascular Ultrasound Doppler [16]. The TBI was calculated by dividing the toe pressure by SBP on limbs of the same side as the literature suggests.

The cut-point values for interpretation of toe pressure and TBI results (i.e., what is considered normal versus low) vary in the literature. This may reflect the lack of evidence regarding the prognostic implication of various TBI cut points. For this study, we considered TBI values of 0.7 or below as normal, 0.3 to 0.7 as mildly low, and less than 0.3 as severely low [17, 18].

A history of lower limb ulceration, including arterial, venous, or mixed arterial and venous aetiology (to feet and lower leg) was captured through patient self-report and review of clinical notes. A history of PAD was based on previous arterial assessments recorded by podiatry and also including any imaging completed by radiographers (including CT scan and/or arterial Doppler) and any clinical notes of relevance entered by a vascular surgeon in the participants medical history and completed as part of standard care.

We introduced measures to control for environmental factors that could potentially influence toe pressure results. The dialysis sites were temperature controlled and set to the same temperature of 20–22°C at all sites. As per current dialysis guidelines each participant attended the dialysis satellite at least 30 minutes before their sessions began. Patients were positioned is a seated position, on a reclining dialysis chair for 20 minutes prior to the first toe pressure assessment [18]. Patients were asked to refrain from drinking coffee during their session but were not asked to refrain from caffeine prior to their attendance.

### Statistical methods

When completing the study protocol there was no previous studies that we could base a sample size on, and as it was a within-subjects study (requiring fewer participants than a between-subject study and allowing for the detection of within person change) [19], a pragmatic sample size of a minimum of 30 participants was selected.

Data was analysed using Stata version 17.0 (StataCorp, College Stations, Texas, USA). A p-value of <0.05 was used to indicate statistical significance. Data was presented using routine descriptive and parametric methods. Specifically, normally distributed results were presented as means (standard deviations), with associated 95% confidence intervals. Comparison of values for each patient was tested for differences using paired t-tests. Linear mixed-effects models were used to test for the effect of patient and clinical factors on change in outcome measures over the five-time points period.

## Results

Thirty-six eligible patients were approached by an investigator and 31 consented to participate. The investigator explained the purpose of the study, confirmed eligibility and gained informed written consent from each participant. Incomplete results were collected (and have been included) for one participant, who was unable to undergo their post-dialysis assessment due to a medical complaint. Patient demographic and clinical variables were collected and are summarised in Table 1. Mean age was 67.4 years, with 16 males (51.6%). Over a third of participants had a previous history of ulceration (38.7%), and two-thirds (67.7%) had diabetes mellitus.

**Table 1:** Patient demographics and clinical characteristics.

	n (%) for categorical values and mean (standard deviation) for continuous values
N	31
Age, mean (SD)	67.4 (11.5)
Male	16 (52%)
Female	15 (48%)
History of ulceration	12 (39%)
Current ulceration	1 (3%)
Prior amputation	3 (10%)
Current peripheral arterial disease (PAD)	2 (7%)
Prior revascularisation for PAD	3 (10%)
Diabetes	21 (68%)
Peripheral neuropathy	9 (29%)
Fluid bolus	1 (3%)
Pre-dialysis weight, mean (SD)	83.1 (23.6)
Post dialysis weight, mean (SD)	81.0 (22.9)
Side of TBI	
Left	11 (36%)
Right	20 (64.5%)

**Fig 1:**
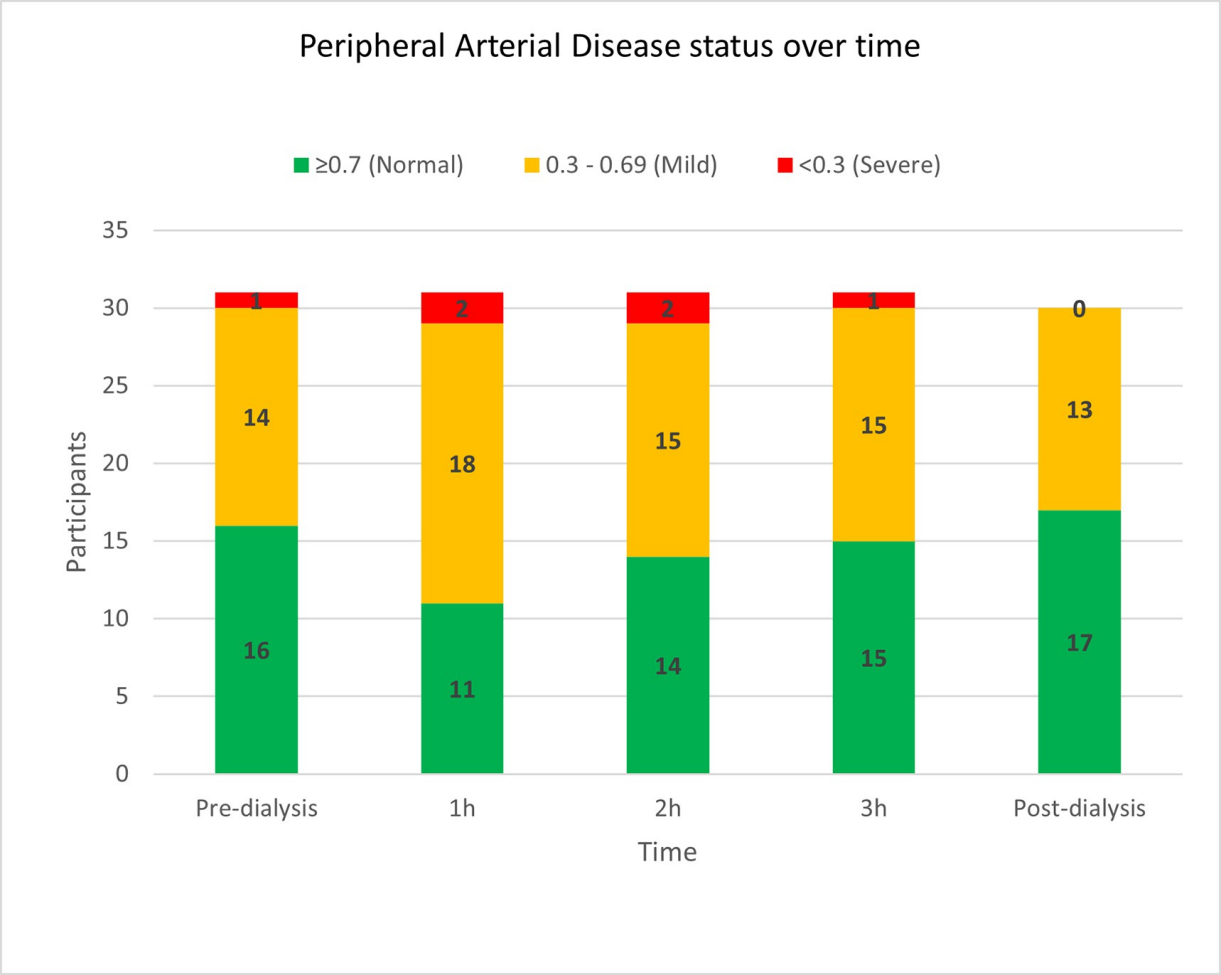
Toe brachial index (TBI) before and during haemodialysis. NB: Incomplete results were collected for one participant, who was unable to complete their fourth hour assessment due to a medical complaint. The last TBI was taken at 3h and was within the ‘Normal’ category’.

Sixteen patients (51.6%) had a normal TBI at baseline, 14 (45.2%) had a mildly low TBI, and only 1 (3.2%) had a severely low TBI (Fig 1) (indicating PAD), which was lower than we expected when compared to the rates reported in the literature) [4]. Fewer patients had normal TBIs at 1 h, 2 h and 3 h timepoints, and there were two patients with a severely low TBI at the 1 h and 2 h timepoints. Between baseline and 1 h, five patients were recategorised from normal to mildly abnormal and one from mildly abnormal to severely abnormal. At the conclusion of haemodialysis (post-dialysis) there were 17 (56.7%) normal TBIs, with no severely abnormal TBIs (p = 0.73). Consistent with this, five of 16 patients with normal TBI (TBI > 0.70) before dialysis were recategorised to mildly-low TBI (TBI 0.30–0.70) and 1 patient with mildly-low TBI was recategorized to severely-low TBI (< 0.30) during dialysis.

Similar trends were observed for mean toe pressure reducing from 111.6 to 94.0 mmHg (p = 0.002) at 1 h and 92.7 mmHg at 2 h (p<0.001). Despite increasing over the remainder of treatment, toe pressures remained at lower mean levels than pre-dialysis values at 3h and post-dialysis (p = 0.016 and p = 0.015, respectively). Mean SBP also declined during dialysis, remaining at lower than pre-dialysis levels throughout (Table 2).

**Table 2:** Vascular characteristics pre-dialysis (baseline), during dialysis (1 hour, 2 hour, 3 hour) and Post-dialysis.

Variable / Time Point	Mean (SD)	95% CI of Mean	Difference in Means (95% CI)^	p-value
**Toe Brachial Index**				
Pre-dialysis	0.72 (0.26)	0.63–0.82	-	-
1 hour	0.63 (0.23)	0.55–0.72	-0.09 (-0.15 to -0.02)	0.01
2 hour	0.64 (0.20)	0.56–0.71	-0.08 (-0.15 to -0.02)	0.01
3 hour	0.69 (0.23)	0.61–0.77	-0.03 (-0.12 to 0.05)	0.41
Post-dialysis	0.73 (0.21)	0.65–0.80	-0.01 (-0.09 to 0.06)	0.68
**Toe Pressure (mmHg, systolic)**				
Pre-dialysis	111.6 (39.1)	97.2–125.9	-	-
1 hour	94.0 (38.2)	80.0–108.0	-17.6 (-28.2 to -7.0)	0.002
2 hour	92.7 (31.3)	81.2–104.2	-18.9 (-28.4 to -9.4)	<0.001
3 hour	97.9 (35.4)	84.9–110.9	-13.7 (-24.6 to -2.7)	0.02
Post-dialysis	103.2 (35.3)	90.0–116.3	-11.2 (-20.0 to -2.3)	0.02
**Systolic Blood Pressure (mmHg, brachial artery)**				
Pre-dialysis	156.7 (23.5)	148.1–165.3	-	-
1 hour	147.5 (23.2)	139.0–156.0	-9.2 (-17.8 to -0.54)	0.04
2 hour	144.8 (16.7)	138.7–150.9	-11.9 (-19.0 to -4.8)	0.002
3 hour	142.1 (23.5)	133.5–150.7	-14.6 (-24.0 to -5.1)	0.004
Post-dialysis	141.5 (22.7)	133.0–150.0	-15.1 (-25.0 to -5.1)	0.004
**Diastolic Blood Pressure (mmHg, brachial artery)**				
Pre-dialysis	74.3 (12.2)	69.8–78.8	-	-
1 hour	70.3 (13.2)	65.5–75.2	-4.0 (-7.8 to -0.2)	0.04
2 hour	69.9 (13.5)	64.9–74.9	-4.4 (-9.2 to 0.3)	0.07
3 hour	70.6 (13.8)	65.3–75.7	-3.7 (-8.2 to 0.7)	0.10
Post-dialysis	70.2 (13.9)	65.2–75.4	-3.8 (-8.6 to 1.0)	0.11

SD–Standard Deviation, CI–Confidence Interval

**Fig 2:**
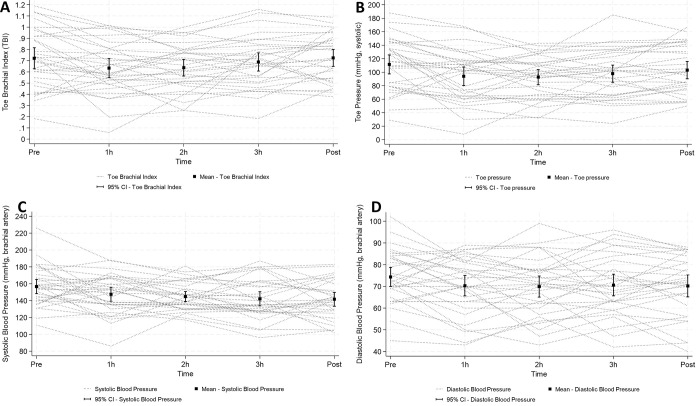
Individual and mean TBI (panel A), toe pressure (B), Systolic Blood Pressure (C) and Diastolic Blood Pressure (D) pre-dialysis (baseline), during dialysis (1 h, 2 h, 3 h) and post-dialysis.

Univariate and multivariable mixed effects model analyses indicate that the trajectory in TBI was impacted by some patient demographic and clinical factors. Mean TBI was higher at baseline in males compared to females (0.86 vs 0.57, p<0.001). Males experienced a fall in TBI during dialysis, however females did not experience a significant change in TBI during dialysis (Fig 2A). Mean TBI was lower in patients with a history of ulceration than in those without (0.81 vs 0.58, p = 0.02), with this difference in mean TBI being maintained at the intra-dialysis timepoints and at p (Fig 2B). Baseline TBI and the change in TBI was similar in patients with a history of PAD compared to those without (Fig 2C).

**Fig 3:**
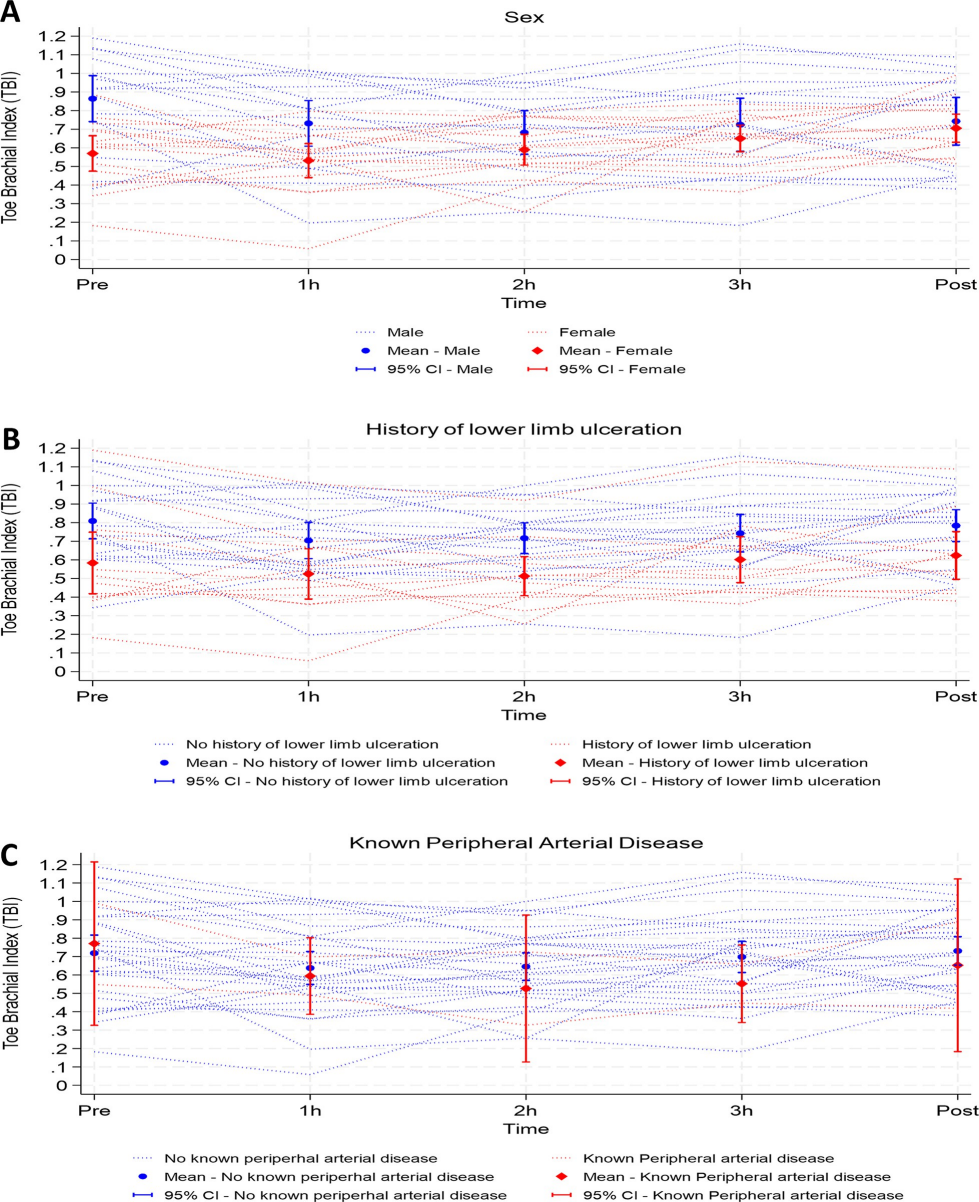
Mean Toe Brachial Index pre-dialysis (baseline), during dialysis (1 h, 2 h, 3 h) and post-dialysis stratified by A. Gender, B. History of lower limb ulceration, C. History of Peripheral Arterial Disease.

Fig 3 reveals the mean TBI across baseline, 1 h, 2 h, 3 h and post-dialysis time points stratified by gender, history of lower limb ulceration and history of PAD. Female participants were more likely to have a lower TBI at baseline (Fig 3A) than males. Patients with a history of foot ulceration had a lower TBI (Fig 3B) compared to than those with no history. Patients with known PAD also experienced a greater average reduction in TBI during dialysis (Fig 3C) than those without.

## Discussion

This is the first study to assess the impact of an entire haemodialysis session on TBI results in people undergoing haemodialysis. This study identified that the mean TBI for participants decreased by the end of the first hour of dialysis from pre-dialysis level, and only returned to this pre-dialysis level at the end of dialysis (post-dialysis). As would be expected given the relationship between toe pressure and TBI, similar trends were seen for absolute toe pressure results at the same time points.

Until recently the variability of TBI and arterial results during haemodialysis has been unknown. A recent study by Carle et al., (2023) similarly looked at the variability of lower limb arterial assessments during haemodialysis and compared pre-dialysis toe pressure and TBI assessments to two timepoints- 1 h and the last 15 minutes of dialysis. They found that whilst toe pressure results dropped significantly, that there was no significant overall change in TBI [13]. Our results may differ given the different timepoints, however this study showed similar changes of both assessments at the same timepoints. In this study the reduction in TBI was mediated by a greater relative decrease in toe pressures, particularly mid-dialysis, attenuated by the falling SBP. Our finding of reducing mean SBPs during haemodialysis is consistent with previous literature [20].

While the overall pre-dialysis mean TBI fell within the normal range, mean TBI at 1 h, 2 h and 3 h was consistent with mild PAD [8]. Similarly, more individuals had a TBI suggesting mild or severe PAD at 1 h, 2 h and 3 h. These results suggest that measuring TBI mid-dialysis may lead to false negatives. This in turn may lead to different management decisions, some of which may not reflect patient need. It is noteworthy that there was inter-patient variability across the sample, including a paradoxical increase in TBI in some patients suggesting conducting TBI during dialysis also leads to false negatives in some patients. This is similar to other studies which have found that individuals with low TBIs may experience inherent inconsistencies between ipsilateral and contralateral readings, which can compromise the validity of TBIs [20].

Our finding that patients with a history of foot ulceration had a lower TBI is consistent with previous studies, which have found an association between reduced TBI, PAD, and adverse outcomes such as foot ulceration, particularly in those with CKD and peripheral neuropathy [21]. In our study, patients with known PAD experienced a greater average reduction in TBI during dialysis than those without. This study was not designed or powered to investigate these differences further. Future research should consider reviewing whether ABI is impacted during haemodialysis in the same way that TBI was and look to conduct a longer follow up to determine if decreases in TBI during dialysis may be a marker of early clinical manifestation of PAD. Previous research looking at TBIs in healthy individuals found that there were no significant differences between men and women [20]. This study suggests that further research also needs to be conducted to determine the potential differences in TBI and potential differences in the pathophysiology of PAD that exist between men and women with kidney failure.

The study findings should be considered in light of certain limitations. First, our sample size consisted of only 31 participants. However, since each participant served as their own control, the variability between participants was reduced, which can lead to increased statistical power and the detection of smaller effects with a smaller sample size. Second, the study was completed across three sites at a single hospital. Third, two podiatrists measured the toe pressures, and this may have led to some differences in technique that may have resulted in inconsistency between raters. We minimised this by using fully automated equipment and having the lead author assess each clinician’s technique prior to the commencement of data collection. Fourth, although guidelines suggest laying patients supine [16] these conditions are not reproducible in a dialysis unit. Thus, patients were tested under the same, reproducible, conditions; in a seated position on a reclining dialysis chair for 20 minutes before testing. Fifth, this study did not compare TBIs and toe pressures to concurrent vascular imaging, or to outcomes such as ulceration, delayed ulcer healing, or amputation to determine the best timepoint to guide further investigation or management. Furthermore, this study did not calculate TBIs on both sides of the body to determine the highest value, as it focused on assessing TBIs on the side of the body opposite to the arteriovenous fistula. Our chosen method of testing the TBI only in the leg corresponding to the arm that was not impacted by the fistula means that there may have been potential that the pressure in the alternate leg may have been different. However, given this is a within subject study, it is unlikely that this would have impacted the results. Sixth, participants may have had hypervolaemia and associated elevated SBP prior to dialysis that may explain the high variability in SBP and TBI when ultrafiltrate was removed, however apart from assessing volume removed to achieve clinically estimated dry weight, volume overload status was not measured as part of this study. Seventh, we did not exclude patients with vasoneural disorders (such as Raynaud’s phenomenon) which may have influenced the results if patients had these conditions. However, as this was a within subject study and participants essentially acted as their own control, it is unlikely that this would have impacted the results. Eighth, our study was also limited to haemodialysis patients only, and no comparison was made to persons not considered high risk (or undergoing haemodialysis) in a seated position for 4–5 hours. It is also important to note that we had a much lower rate of known PAD in our recruited sample than we expected (6% versus 18%-31% reported in the literature) and so our results may reflect that of a healthier population than comparable populations receiving haemodialysis. This may in part be due to local hospital procedures that prioritise revascularisation of compromised patients (a further 9% of participants had undergone a procedure and were no longer considered compromised).

### Clinical Implications

The findings from this study suggest that TBI and other clinical vascular assessments should be completed either before, or ideally between, dialysis sessions to minimise intradialytic changes and subsequent inaccurate results that might lead to inappropriate clinical decision making.

## Conclusion

TBI and toe pressures are significantly impacted by dialysis, and results vary greatly. This study identified that during haemodialysis the mean TBI results decreased during dialysis and only returned to baseline at the completion of dialysis. Clinicians should be mindful that mean TBI and mean toe pressures are impacted by haemodialysis and that these assessments should not be undertaken during dialysis, and that treatments that include sharp debridement and moist wound healing should not be decided based off intradialytic arterial assessment results. To enhance the potential value of TBI as a metric, there is a need to prioritise adherence to established protocols and explore methods to more effectively assess vascular supply to the foot.

## Supporting information

S1 Checklist Human participants research checklist. (DOCX)

S1 File TBI in ESKD. (PDF)

S1 Dataset TBI is ESKD. (XLSX)
