# Supplementary material for: Temporal changes in toe-brachial index results in haemodialysis patients
Source: PLoS One. 2024 Apr 25;19(4):e0301376. doi: 10.1371/journal.pone.0301376 (PMC11045130; doi:10.1371/journal.pone.0301376)
Supplement: S1 File — (PDF) [file pone.0301376.s002.pdf]

Northern Health

# Research Protocol Template (Low Risk Research Project and Clinical Audits)

A well written and complete protocol is essential for a high quality research project. A study protocol generally follows a conventional layout. There are several templates already available although most are developed for commercially-sponsored randomised controlled studies. This research protocol template aims to offer Northern Health surgical medical, nursing, midwifery and allied health researchers a more generic guide suitable for a broader range of scientific research enquiries.

The preparation of a protocol is an important first step in the research process for the following reasons:

1. It states the research question you aim to answer;
2. It encourages adequate consideration and planning of project detail *before* you begin;
3. It allows co-investigators or peers a living and dynamic document for contribution and early review prior to its completion;
4. It acts as a record and reminder for you and your supervisor (co-investigator or co-worker) of the initial project aims and stated procedures. This record also enables you to monitor the progress of your project; and
5. It provides the basis for funding or human research ethics applications.

When drafting your protocol:

- Discuss project design and scope with supervisors/peers.
- Discuss appropriate statistical analysis methods with a statistician.
- Delete sections that are not applicable.
- Add version number and document date in the footer.

Northern Health

# The association between toe-brachial index results and haemodialysis in patients with End Stage Kidney Disease

## STUDY INVESTIGATOR(S)

### Principal Investigator


| | | |
|---|---|---|
| Title and Name | Belinda Baines | |
| Appointment/s | Grade 2 Podiatrist | |
| Department / Affiliation with Northern Health | Allied Health- Podiatry and Orthotics | |
| Qualifications | Bachelor of Health Science, Master of Podiatric Practice | |
| Phone | 03 8405 8017 | Fax: |
| Mobile/ Pager | 0422019277 | Email: belinda.baines@nh.org.au |
| Is this person the contact person for the project? | Yes / No (please circle / highlight) | |


### Associate Investigator


| | | |
|---|---|---|
| Title and Name | Dr Rebecca Jessup | |
| Appointment/s | Allied Health Research Lead | |
| Department/ Affiliation with Northern Health | Allied Health | |
| Qualifications | PhD, MPH, B.Pod | |
| Phone | 03 8468 0737 | |
| Email: | Rebecca.jessup@nh.org.au | |


### Associate Investigator


| | | |
|---|---|---|
| Title and Name | Dr Matthew Cotchett | |
| Appointment/s | Grade 3 Advanced Practicing Podiatrist | |
| Department/ Affiliation with Northern Health | Allied Health- Podiatry and Orthotics | |
| Qualifications | B.Sc., B.Pod (Hons)., PhD | |
| Phone | 03 8405 8017 | |
| Email: | matthew.cotchett@nh.org.au | |

**Associate Investigator**

| | | |
|---|---|---|
| Title and Name | Mr Mark Tacey | |
| Appointment/s | Biostatistician | |
| Department/ Affiliation with Northern Health | Northern Health Office of Research | |
| Qualifications | MBiostat, BSc | |
| Phone | 8468 0742 | Mobile |
| Email: | Mark.Tacey@nh.org.au | |

**Associate Investigator**

| | | |
|---|---|---|
| Title and Name | Dr Timothy Pianta | |
| Appointment/s | Nephrologist | |
| Department/ Affiliation with Northern Health | Physician- Nephrology and Obstetric Medicine | |
| Qualifications | MBBS (Hons), PhD, FRACP | |
| Phone | 84058000 | Mobile |
| Email: | Timothy.Pianta@nh.org.au | |

*Note: Copy and paste the above table if there are more investigators on this study.*

## 1. INTRODUCTION

Current standard practice for lower limb arterial assessment to determine the presence of peripheral arterial disease includes the use of a toe brachial index (TBI), whereby the toe systolic blood pressure is divided by the brachial systolic blood pressure. Anecdotal evidence suggests that due to patient convenience clinicians often assess TBIs during haemodialysis. Historically this has not taken into consideration the fact that those with CKD on haemodialysis can experience changes in their systolic blood pressure during dialysis, which is known as intradialytic hypertension and intradialytic hypotension. These changes may in turn affect the results of toe brachial index.

Currently it is not known if dialysis affects toe systolic pressures. This study will aim to monitor both brachial and toe systolic blood pressures to obtain TBI results, with measurements taken at specific time intervals (pre-dialysis, during dialysis (hourly) and post-dialysis) to monitor the association between the two. These results may then be applied to clinical practice to determine the suitability of completing TBI assessments during dialysis. This information will guide clinical practice, as these assessments guide decisions regarding sharp debridement, and wound dressing selection.

## 2. BACKGROUND

Individuals with chronic kidney disease (CKD), including those with end-stage kidney disease (ESKD) receiving haemodialysis treatment are at inordinate risk of lower limb ulcerations, infection, and amputations compared to individuals in the general population with normal

renal function (Ishioka et al., 2018). For patients on dialysis - apart from a history of prior ulceration or amputation - the greatest risk factors for ulceration for are peripheral arterial disease (PAD), neuropathy, and cerebrovascular disease (Kaminski 2019). Peripheral arterial disease is highly prevalent in ESKD. Patients with CKD often have one or more risk factors associated with PAD such as older age, smoking, diabetes, hypertension and hyperlipidemia. CKD itself imposes greater additional risk due to chronic inflammation, hypoalbuminemia, and a pro-calcific state (Garimella et al, 2014). Almost all patients with end-stage kidney disease have evidence of peripheral neuropathy, although clinical burden is greatest in those with concurrent diabetes (Arnold 2017).There is a need to identify patients at risk of ulceration and amputation, and when ulceration occurs prevent progression, amputation or re-ulceration.
According to the International Working Group on the Diabetic Foot guidelines on Peripheral Arterial Disease (https://iwgdfguidelines.org/wp-content/uploads/2019/05/04-IWGDF-PAD-guideline-2019.pdf ) first line assessment to determine the presence of PAD is to compile a detailed relevant medical history and palpate pedal pulses. For patients in high risk groups, these steps alone are not reliable enough to exclude PAD, and subsequent screening assessments should be performed (Hinchcliff et al., 2019). This may include evaluation of pedal Doppler arterial waveforms in combination with performing an Ankle Brachial Index (ABI) or toe brachial index (TBI). ABIs are calculated by measuring the systolic blood pressure at the ankle and dividing it by the systolic blood pressure at the arm. Alternatively, TBIs are calculated by measuring the systolic blood pressure at a toe and dividing it by the systolic blood pressure at the arm.

The ABI has been shown to be both specific and sensitive in the diagnosis of PAD (Hinchcliff et al., 2019) however in some patients it may yield a falsely elevated result due to the presence of medial artery calcification, a condition common to people in the advanced stages of both diabetes and CKD. In this case, it is preferable to measure toe systolic pressure and conduct a TBI. There is no single measure that has been shown to provide an optimal assessment of risk of PAD, however PAD is less likely when an ABI is between 0.9-1.3; toe pressure measurements are higher than 30mmHg; or when a TBI is higher than 0.75, and Doppler waveforms are triphasic. Using more than one of these tests in parallel enables clinicians to make a more accurate diagnosis. Recent research has suggested that the use of pedal Doppler arterial waveforms in combination with TBI is the superior lower limb arterial assessment (Brownrigg et al., 2016). Clinical guidelines recommend that patients undergoing ABI, TBI and audible doppler assessments are seated for at least 20 minutes prior to assessment (Hinchcliff et al., 2019).

Expert evaluation for PAD including use of TBI is also standard-of-care in the management of foot ulcers in high-risk patients such as those with ESKD. PAD can alter the body's ability to heal a foot ulceration, and can lead to infection, increased tissue loss and insufficient delivery of nutrition, oxygen and antibiotics, all of which can lead to further foot amputation (Amin and Doupis, 2016). The presence of PAD needs to be promptly excluded so that clinicians can safely perform adequate ulcer treatment including sharp debridement to assist with wound preparation. It is also necessary to have this information in order to decide whether a dry or moist wound dressing is suitable to treat a DFU (Roesing and Andrews, 2019).

Due to the great demands on patient times of haemodialysis, it is common for podiatry assessment and treatment to occur opportunistically during dialysis sessions. However, there has been no systematic evaluation of the impact of haemodialysis and in particular changes in blood pressure during dialysis may affect the measurement of TBI. Blood pressure is routinely measured before, during and after haemodialysis and patients may experience an fall in blood pressure during or immediately after haemodialysis known as intradialytic hypotension (Sars B et al., 2020) or a paradoxical increase in blood pressure known as intradialytic hypertension (Van Buren and Inrig, 2016). Pre-dialysis systolic blood pressure is typically around 10mmHg higher than intradialytic blood pressure and post-dialysis systolic blood pressure is around 10mmHg less than intradialytic blood pressure (Sterns et al., 2014). When intradialytic hypertension occurs, it is most common in the second or third hour of hemodialysis, and after significant ultrafiltration has taken place (Inrig, 2009). During dialysis blood is also diverted to an extracorporeal circuit, which may result in contraction of blood vessels to maintain blood pressure.

The results of this research will aim to assist clinicians with clinical guidelines around arterial assessments and also wound management for those undergoing haemodialysis.

## 3. AIM(S) AND OBJECTIVES OF STUDY

The aim of this study is to evaluate toe brachial indices, during haemodialysis, in participants with end stage kidney disease.

## 4. RESEARCH PLAN/METHODOLOGY

### 4.1 Participants

Selection criteria:
30 participants with chronic kidney disease undergoing haemodialysis will be recruited from Northern Health Dialysis Satellite Clinics at Northern Hospital Epping, Craigieburn Centre and Broadmeadows Hospital. Participants will have been admitted as same-day inpatients. Participants will be excluded if they have cognitive impairment or are under 18 years of age. Participants speaking any language will be included and interpreters used as required.

Participant/data (de-)identification procedures:
Identifiable patient data will be replaced with a code/unique number. The master list of names and matching codes will be stored electronically and password protected by the PI.

### 4.2 Measures

The primary outcomes for this study will be toe brachial index, measured pre-dialysis, during dialysis (at 1, 2, and 3 hours), and post-dialysis.

Additional measures will include routinely collected clinical data:

- each timepoint: ultrafiltration (fluid-removal) rate, and dialysis blood flow rate

- Each session: pre- and post-dialysis weight, requirement for fluid bolus for symptomatic intradialytic hypotension
- Each subject – demographic data, history of current ulceration, prior amputation, prior ulceration, PVD, revascularization for PVD, diabetes, diagnosed peripheral neuropathy.

## 4.3 Procedures

Consent process(es):
All participants to be consented will be directly approached by the PI (BB) to participate in the study. The PI or one of the research assistants will arrange a screening for eligibility with each patient and arrange data collection on a suitable day that the participant will be undergoing dialysis at a NH satellite clinic. Once the participant has been informed and verbally consented to participate, written information and a consent form will be provided.

Details of data collection, processing and analysis :
Patients attending NH dialysis service will be approached over six months until 30 participants are recruited. The PI or research assistant will only approach those patients who are determined by the dialysis nurses as being clinically appropriate to participate based on the inclusion criteria. Written, informed consent will be obtained prior to each assessment by a study investigator who is an employee of NH (BB).

TBIs will completed on each participant pre-dialysis, at hourly intervals during dialysis, and post-dialysis.

Record keeping procedures, including storage of data access and destruction:
The patient data will be kept strictly confidential according to the National Statement on Ethical Conduct in Human Research 2007 and the Australian Code for Responsible Conduct of Research 2007.

- Consent forms will be scanned into CPF under admission date. The hard-copy version will be kept by the PI and research team in the Foot Procedure Room (key access required) in a locked cupboard and a copy will be given to participants.
- All recorded data will be saved on a NH password protected computer, and will only be accessed by named investigators.
- Patients who participate will be re-identifiable by linking de-identified information with an 'auxiliary dataset' that contains identifying information. The de-identified information and the auxiliary dataset will be kept in separate password encrypted files on Northern Health shared drive.Patient data will be only be transferred and analysed in a coded form.
- Individual patients will not be identifiable from the presented or published material.

## 4.4 STATISTICAL CONSIDERATIONS

The sample size has not been able to be guided based on published information, as no details relating to the variance from patient to patient in the change in toe brachial indices over time can be found.  Stern (2014) indicates that the pre-dialysis and post-dialysis systolic

blood pressure are found to be 10 mm Hg more and 10 mm Hg less than the interdialytic blood pressure respectively [13], but there is no indication of the variance from patient to patient in the change in toe brachial indices over time. Thus, the sample size of n=30 patients has been selected as being able to be conducted effectively within the timeframe of the study. This will then indicate if additional sample is required to address the needs of the study.

Descriptive analysis will be conducted to summarise the toe brachial index results over the study timepoints for each patient. Data will be presented as means (standard deviations) if normally distributed or as median (inter-quartile ranges) if skewed. The mean of changes from one timepoint to the next are likely to be normally distributed, and thus the data will be assessed using paired t-tests. More detailed analysis, including longitudinal analysis using mixed-effects linear regression models may be explored if the sample size allows an investigation of the effects of some patient characteristics. A p-value of less than 0.05 will indicate statistical significance, with the data to be compiled in Microsoft Excel and analysed using Stata version 15.1 (StataCorp, College Stations, Texas, USA).

### 4.5 ETHICAL CONSIDERATIONS

The study will be conducted according to the NHMRC National Statement on Ethical Conduct in Human Research (2007 and updates).
This study will then be prepared for research publication and presentation at an appropriate scientific conference.

## 5. REFERENCES

Amin, N., & Doupis, J. (2016). Diabetic foot disease: From the evaluation of the "foot at risk" to the novel diabetic ulcer treatment modalities. *World journal of diabetes*, *7*(7), 153–164. https://doi.org/10.4239/wjd.v7.i7.153

Arnold, R., Pianta, T., Pussell, B., Kirby, A., &O'Brien, K. (2017) Randomized, controlled trial of the effect of dietary potassium restriction on nerve function in CKD. Clinical Journal of the American Society of Nephrology

Brownrigg, J. R. W., Hinchliffe, R. J., Apelqvist, J., Boyko, E. J., Fitridge, R., Mills, J. L., Reekers, J., Shearman, C. P., Zierler, R. E., Schaper, N. C., and on behalf of the International Working Group on the Diabetic Foot (IWGDF) ( 2016) Effectiveness of bedside investigations to diagnose peripheral artery disease among people with diabetes mellitus: a systematic review. *Diabetes Metab Res Rev*, 32: 119– 127. doi: 10.1002/dmrr.2703.

Garimella, P. S., & Hirsch, A. T. (2014). Peripheral artery disease and chronic kidney disease: clinical synergy to improve outcomes. *Advances in chronic kidney disease*, *21*(6), 460–471. https://doi.org/10.1053/j.ackd.2014.07.005

Hinchcliffe, R., Forsythe, R., Apelqvist, J., Boyko, E., Fitridge, R., & Hong, J. et al. (2019). IWGDF Guileline on diagnosis, poognosis and management of peripheral artery disease in patients with a foot ulcer and diabetes. Retrieved 28 February 2020, from

https://iwgdfguidelines.org/wp-content/uploads/2019/05/04-IWGDF-PAD-guideline-2019.pdf

Inrig J. K. (2010). Intradialytic hypertension: a less-recognized cardiovascular complication of hemodialysis. *American journal of kidney diseases : the official journal of the National Kidney Foundation*, *55*(3), 580–589. https://doi.org/10.1053/j.ajkd.2009.08.013

Ishioka, K., Ohtake, T., Moriya, H. *et al.* High prevalence of peripheral arterial disease (PAD) in incident hemodialysis patients: screening by ankle-brachial index (ABI) and skin perfusion pressure (SPP) measurement. *Ren Replace Ther* **4,** 27 (2018). https://doi.org/10.1186/s41100-018-0168-5

Kaminski, M.R., Lambert, K.A., Raspovic, A. *et al.* Risk factors for foot ulceration in adults with end-stage renal disease on dialysis: a prospective observational cohort study. *BMC Nephrol* **20,** 423 (2019). https://doi.org/10.1186/s12882-019-1594-5

Roesing T.L., Andrews J. (2019) The Diagnosis and Treatment of Common Wounds Encountered in Primary Care. In: Russell J., Ryan Jr. E. (eds) Common Dermatologic Conditions in Primary Care. Current Clinical Practice. Humana, Cham

Sars B, van der Sande F, M, Kooman J, P: Intradialytic Hypotension: Mechanisms and Outcome. Blood Purif 2020;49:158-167. doi: 10.1159/000503776

Stern, A., Sachdeva, S., Kapoor, R., Singh, J., & Sachdeva, S. (2014). High blood pressure in dialysis patients: cause, pathophysiology, influence on morbidity, mortality and management. *Journal of clinical and diagnostic research : JCDR*, *8*(6), ME01–ME4. https://doi.org/10.7860/JCDR/2014/8253.4471

Van Buren, P. N., & Inrig, J. K. (2016). Mechanisms and Treatment of Intradialytic Hypertension. *Blood purification*, *41*(1-3), 188–193. https://doi.org/10.1159/000441313
